# DO POSITIVE AFFECT AND LONELINESS PREDICT LONGEVITY ACROSS CULTURES? FINDINGS FROM THE UNITED STATES AND JAPAN

**DOI:** 10.1093/geroni/igad104.1581

**Published:** 2023-12-21

**Authors:** Takeshi Nakagawa, Gizem Hueluer, Andrea Horn

**Affiliations:** National Center for Geriatrics and Gerontology, Obu, Aichi, Japan; University of Bonn, Bonn, Nordrhein-Westfalen, Germany; University of Zurich, Zurich, Zurich, Switzerland

## Abstract

Prior studies have revealed the beneficial effects of positive affect and the harmful effects of negative affect on health and longevity. The majority of the evidence comes from Western countries, but cross-cultural studies suggest that the relationship between affect and health varies by culture. In this preregistered secondary analysis (https://osf.io/ke5pr), we investigated variations in the links of positive affect and loneliness with longevity across cultures. Data were derived from comparable surveys in the United States (1986–2005) and Japan (1987–2006). We identified case-matched American and Japanese samples based on age and sex (n = 1,663 per country, mean age = 70 years). Positive affect (2 items), negative affect (2 items), and loneliness (1 item) were measured using five available items from the Center for Epidemiological Studies Depression Scale. The measurement model of positive and negative affect was invariant across cultures. The affect scores were standardized based on each sample. Cox proportional hazard models assessed the associations of positive affect and loneliness with mortality. We used multiple imputation for missing data. Greater positive affect was associated with reduced mortality risk across cultures (hazard ratio [95% confidence interval] = 0.955 [0.914–0.998]) after controlling for negative affect and demographic and health factors. Culture did not moderate the association. Additional adjustment for behavioral factors attenuated the hazard ratio to statistical non-significance. Loneliness and negative affect were not associated with mortality. Our findings suggest that the health benefits of positive affect are primarily similar across cultures.

